# 3,3-Dibromo-1-ethyl-1*H*-2,1-benzo­thiazin-4(3*H*)-one 2,2-dioxide

**DOI:** 10.1107/S1600536808017510

**Published:** 2008-06-13

**Authors:** Muhammad Shafiq, M. Nawaz Tahir, Islam Ullah Khan, Saeed Ahmad, Waseeq Ahmad Siddiqui

**Affiliations:** a Department of Chemistry, Government College University, Lahore, Pakistan; b Department of Physics, University of Sargodha, Sagrodha, Pakistan; c Department of Chemistry, University of Science and Technology Bannu, Bannu, Pakistan; d Department of Chemistry, University of Sargodha, Sagrodha, Pakistan

## Abstract

In the mol­ecule of the title compound, C_10_H_9_Br_2_NO_3_S, the S atom is four-coordinated in distorted tetra­hedral configuration. The heterocyclic thia­zine ring adopts a twist conformation. An intra­molecular C—H⋯O hydrogen bond results in the formation of a non-planar five-membered ring. In the crystal structure, inter­molecular C—H⋯O hydrogen bonds link the mol­ecules into infinite chains along the *c* axis.

## Related literature

For related literature, see: Franzén (2000 ▶); Misu & Togo (2003 ▶); Shafiq *et al.* (2008 ▶); Tahir *et al.* (2008 ▶). For ring puckering parameters, see: Cremer & Pople (1975 ▶).
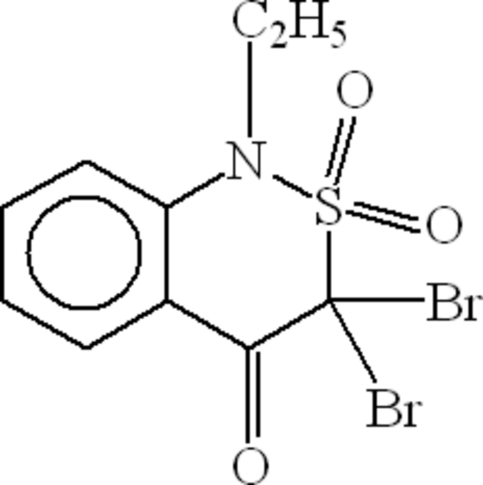


## Experimental

### 

#### Crystal data

C_10_H_9_Br_2_NO_3_S
*M* *_r_* = 383.06
Monoclinic, 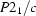

*a* = 7.7979 (5) Å
*b* = 11.9645 (7) Å
*c* = 13.1231 (8) Å
β = 95.374 (3)°
*V* = 1218.98 (13) Å^3^
*Z* = 4
Mo *K*α radiation
μ = 6.82 mm^−1^
*T* = 296 (2) K
0.15 × 0.12 × 0.10 mm

#### Data collection

Bruker Kappa APEXII CCD diffractometer
Absorption correction: multi-scan (*SADABS*; Bruker, 2005 ▶) *T*_min_ = 0.400, *T*_max_ = 0.508
14754 measured reflections
3281 independent reflections
1708 reflections with *I* > 2σ(*I*)
*R*_int_ = 0.059

#### Refinement

*R*[*F* ^2^ > 2σ(*F* ^2^)] = 0.066
*wR*(*F* ^2^) = 0.221
*S* = 1.02
3281 reflections
154 parameters
H-atom parameters constrained
Δρ_max_ = 1.27 e Å^−3^
Δρ_min_ = −1.61 e Å^−3^

### 

Data collection: *APEX2* (Bruker, 2007 ▶); cell refinement: *APEX2*; data reduction: *SAINT* (Bruker, 2007 ▶); program(s) used to solve structure: *SHELXS97* (Sheldrick, 2008 ▶); program(s) used to refine structure: *SHELXL97* (Sheldrick, 2008 ▶); molecular graphics: *ORTEP-3 for Windows* (Farrugia, 1997 ▶) and *PLATON* (Spek, 2003 ▶); software used to prepare material for publication: *WinGX* (Farrugia, 1999 ▶) and *PLATON*.

## Supplementary Material

Crystal structure: contains datablocks global, I. DOI: 10.1107/S1600536808017510/hk2472sup1.cif

Structure factors: contains datablocks I. DOI: 10.1107/S1600536808017510/hk2472Isup2.hkl

Additional supplementary materials:  crystallographic information; 3D view; checkCIF report

## Figures and Tables

**Table 1:** Hydrogen-bond geometry (Å, °)

*D*—H⋯*A*	*D*—H	H⋯*A*	*D*⋯*A*	*D*—H⋯*A*
C2—H2⋯O1^i^	0.93	2.52	3.390 (10)	157
C9—H9*A*⋯O1	0.97	2.38	2.876 (11)	111

Symmetry code: (i) 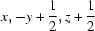.

**Fig. 1.**
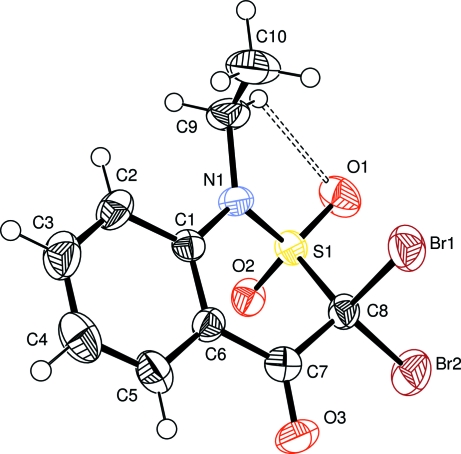
The molecular structure of the title molecule, with the atom-numbering scheme. Displacement ellipsoids are drawn at the 50% probability level. Hydrogen bond is shown as dashed line.

**Fig. 2.**
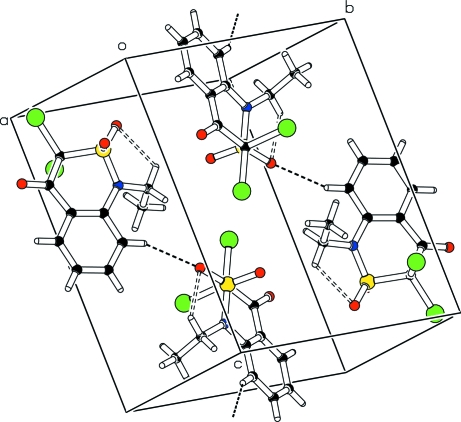
A packing diagram of (I). Hydrogen bonds are shown as dashed lines.

## supplementary crystallographic information

### Comment

The synthesis of heterocyclic system is of continuing interest because a large number of biologically active molecules contain heterocyclic rings (Franzén, 2000). 2,1-Benzothiazine is a relatively unexplored ring system with respect to both its synthesis and biological activity, in which it belongs to an important heterocyclic class of compounds, although it finds a number of applications in medicinal chemistry. The derivatives of 2,1-benzothiazine are used as drugs for heart diseases and also show lipoxygenase inhibition activity (Misu & Togo, 2003). Recently we have reported the crystal structures of 1-ethyl-1*H* -2,1-benzothiazin-4(3*H*) one 2,2-dioxide, (II) (Shafiq *et al.*, 2008) and 1-methyl-1*H*-2,1-benzothiazin-4(3*H*) one 2,2-dioxide, (III) (Tahir *et al.*,  2008), in which they contain the same heterocyclic ring. We report herein the syntesis and crystal structure of the title compound, (I), which is obtained from the bromination of (II).

In the molecule of (I) (Fig. 1), the bond lengths and angles are within normal ranges, which are comparable with the corresponding values in (II). The S1-N1 [1.617 (6) Å], S1-C8 [1.792 (8) Å] and C7-C8 [1.540 (11) Å] bonds in (I) are reported as 1.6405 (15), 1.750 (2) and 1.510 (3) Å, respectively, in (II). The S atom is four-coordinated in distorted tetrahedral configuration (Table 1) by one N and one C atoms of the heterocyclic ring and two O atoms. Ring A (C1-C6) is, of course, planar, and it is oriented with respect to (S1/O1/O2) and (C8/Br1/Br2) planes at dihedral angles of 78.44 (32)° and 77.79 (28)°, respectively. Ring B (S1/N1/C1/C6-C8) adopts twisted conformation, having total puckering amplitude, Q_T_, of 0.763 (2) Å (Cremer & Pople, 1975). The intra- molecular C-H···O hydrogen bond (Table 2) results in the formation of a non-planar five-membered ring C (O1/S1/N1/C9/H9A).

In the crystal structure, intermolecular C-H···O hydrogen bonds (Table 2) link the molecules into infinine chains along the c axis (Fig. 2), in which they may be effective in the stabilization of the structure.

### Experimental

Compound (I) was prepared by the reaction of (II) (34.0 mg, 0.15 mmol), *N*-bromosuccinimide (57.0 mg, 0.32 mmol) and dibenzoyl peroxide (2.1 mg, 0.009 mmol) in CCl_4_ (8 ml) by heating under reflux for 2 h. Crystals suitable for X-ray analysis were obtained by evaporating the solvent slowly at room temperature for about 7 d (m.p. 394-395 K).

### Refinement

The highest peak and deepest hole in the final difference electron density map are located 1.27 and 1.61 Å from Br1 and Br2 atoms, respectively. H atoms were positioned geometrically, with C-H = 0.93, 0.97 and 0.96 Å for aromatic, methylene and methyl H and constrained to ride on their parent atoms with U_iso_(H) = xU_eq_(C), where x = 1.5 for methyl H and x = 1.2 for all other H atoms.

### Crystal data

**Table:**

C_10_H_9_Br_2_NO_3_S	*F*_000_ = 744
*M**_r_* = 383.06	*D*_x_ = 2.087 Mg m^−^^3^
Monoclinic, *P*2_1_/*c*	Mo *K*α radiation λ = 0.71073 Å
Hall symbol: -P 2ybc	Cell parameters from 708 reflections
*a* = 7.7979 (5) Å	θ = 2.3–29.2º
*b* = 11.9645 (7) Å	µ = 6.82 mm^−^^1^
*c* = 13.1231 (8) Å	*T* = 296 (2) K
β = 95.374 (3)º	Prismatic, red
*V* = 1218.98 (13) Å^3^	0.15 × 0.12 × 0.10 mm
*Z* = 4	

### Data collection

**Table:**

Bruker KappaAPEXII CCD diffractometer	3281 independent reflections
Radiation source: fine-focus sealed tube	1708 reflections with *I* > 2σ(*I*)
Monochromator: graphite	*R*_int_ = 0.059
Detector resolution: 7.40 pixels mm^-1^	θ_max_ = 29.2º
*T* = 296(2) K	θ_min_ = 2.3º
ω scans	*h* = −8→10
Absorption correction: multi-scan(SADABS; Bruker, 2005)	*k* = −16→15
*T*_min_ = 0.400, *T*_max_ = 0.508	*l* = −17→18
14754 measured reflections	

### Refinement

**Table:**

Refinement on *F*^2^	Secondary atom site location: difference Fourier map
Least-squares matrix: full	Hydrogen site location: inferred from neighbouring sites
*R*[*F*^2^ > 2σ(*F*^2^)] = 0.066	H-atom parameters constrained
*wR*(*F*^2^) = 0.221	*w* = 1/[σ^2^(*F*_o_^2^) + (0.1081*P*)^2^ + 4.0099*P*] where *P* = (*F*_o_^2^ + 2*F*_c_^2^)/3
*S* = 1.03	(Δ/σ)_max_ < 0.001
3281 reflections	Δρ_max_ = 1.27 e Å^−^^3^
154 parameters	Δρ_min_ = −1.61 e Å^−^^3^
Primary atom site location: structure-invariant direct methods	Extinction correction: none

### Special details

**Table:**

Geometry. All esds (except the esd in the dihedral angle between two l.s. planes) are estimated using the full covariance matrix. The cell esds are taken into account individually in the estimation of esds in distances, angles and torsion angles; correlations between esds in cell parameters are only used when they are defined by crystal symmetry. An approximate (isotropic) treatment of cell esds is used for estimating esds involving l.s. planes.
Refinement. Refinement of F^2^ against ALL reflections. The weighted R-factor wR and goodness of fit S are based on F^2^, conventional R-factors R are based on F, with F set to zero for negative F^2^. The threshold expression of F^2^ > 2sigma(F^2^) is used only for calculating R-factors(gt) etc. and is not relevant to the choice of reflections for refinement. R-factors based on F^2^ are statistically about twice as large as those based on F, and R- factors based on ALL data will be even larger.

### Fractional atomic coordinates and isotropic or equivalent isotropic displacement parameters (Å^2^)

**Table:**

	*x*	*y*	*z*	*U*_iso_*/*U*_eq_	
Br1	0.97364 (12)	0.08723 (8)	0.18987 (7)	0.0520 (3)	
Br2	0.71557 (15)	−0.05878 (9)	0.04712 (7)	0.0616 (4)	
S1	0.5854 (3)	0.09863 (16)	0.20346 (14)	0.0353 (5)	
O1	0.5802 (9)	0.1912 (5)	0.1362 (5)	0.0554 (17)	
O2	0.4378 (7)	0.0280 (5)	0.2029 (4)	0.0427 (14)	
O3	0.7951 (9)	−0.1813 (5)	0.2399 (5)	0.0521 (16)	
N1	0.6503 (9)	0.1398 (5)	0.3180 (5)	0.0346 (15)	
C1	0.6964 (10)	0.0579 (6)	0.3926 (6)	0.0296 (16)	
C2	0.6905 (11)	0.0854 (7)	0.4974 (6)	0.0405 (19)	
H2	0.6569	0.1565	0.5163	0.049*	
C3	0.7351 (12)	0.0054 (9)	0.5708 (7)	0.051 (2)	
H3	0.7337	0.0242	0.6395	0.062*	
C4	0.7809 (13)	−0.0996 (8)	0.5460 (7)	0.054 (2)	
H4	0.8088	−0.1523	0.5970	0.064*	
C5	0.7859 (11)	−0.1275 (7)	0.4450 (6)	0.044 (2)	
H5	0.8149	−0.2001	0.4278	0.053*	
C6	0.7480 (10)	−0.0488 (6)	0.3675 (6)	0.0319 (16)	
C7	0.7694 (10)	−0.0849 (6)	0.2634 (6)	0.0347 (17)	
C8	0.7569 (10)	0.0054 (7)	0.1794 (6)	0.0348 (17)	
C9	0.6490 (12)	0.2600 (6)	0.3460 (7)	0.047 (2)	
H9A	0.5998	0.3023	0.2873	0.056*	
H9B	0.5745	0.2700	0.4005	0.056*	
C10	0.8162 (14)	0.3057 (8)	0.3789 (9)	0.065 (3)	
H10A	0.8047	0.3832	0.3958	0.098*	
H10B	0.8902	0.2984	0.3248	0.098*	
H10C	0.8651	0.2657	0.4381	0.098*	

### Atomic displacement parameters (Å^2^)

**Table:**

	*U*^11^	*U*^22^	*U*^33^	*U*^12^	*U*^13^	*U*^23^
Br1	0.0447 (6)	0.0622 (6)	0.0509 (6)	−0.0139 (4)	0.0134 (4)	−0.0031 (4)
Br2	0.0757 (8)	0.0722 (7)	0.0374 (5)	−0.0052 (5)	0.0077 (5)	−0.0101 (4)
S1	0.0405 (12)	0.0358 (10)	0.0293 (10)	0.0021 (9)	0.0010 (8)	0.0063 (8)
O1	0.069 (4)	0.048 (3)	0.049 (4)	0.003 (3)	−0.001 (3)	0.021 (3)
O2	0.032 (3)	0.053 (4)	0.043 (3)	−0.010 (3)	0.005 (3)	−0.007 (3)
O3	0.074 (5)	0.035 (3)	0.047 (4)	0.009 (3)	0.010 (3)	−0.009 (3)
N1	0.049 (4)	0.026 (3)	0.028 (3)	0.002 (3)	0.000 (3)	−0.001 (2)
C1	0.027 (4)	0.031 (4)	0.030 (4)	−0.006 (3)	0.000 (3)	−0.002 (3)
C2	0.041 (5)	0.050 (5)	0.031 (4)	−0.003 (4)	0.001 (4)	−0.012 (4)
C3	0.057 (6)	0.066 (6)	0.032 (4)	−0.012 (5)	0.005 (4)	−0.005 (4)
C4	0.056 (6)	0.060 (6)	0.043 (5)	−0.004 (5)	−0.003 (4)	0.020 (4)
C5	0.046 (5)	0.044 (5)	0.044 (5)	0.000 (4)	0.009 (4)	0.014 (4)
C6	0.030 (4)	0.034 (4)	0.033 (4)	−0.001 (3)	0.006 (3)	−0.004 (3)
C7	0.031 (4)	0.035 (4)	0.038 (4)	0.001 (3)	0.000 (3)	0.000 (3)
C8	0.037 (5)	0.040 (4)	0.027 (4)	−0.008 (4)	0.004 (3)	−0.004 (3)
C9	0.058 (6)	0.026 (4)	0.056 (5)	0.013 (4)	0.005 (4)	0.000 (4)
C10	0.069 (7)	0.036 (5)	0.091 (8)	−0.011 (5)	0.008 (6)	−0.011 (5)

### Geometric parameters (Å, °)

**Table:**

Br1—C8	1.947 (8)	C3—H3	0.9300
Br2—C8	1.898 (7)	C4—C5	1.371 (13)
S1—O1	1.415 (6)	C4—H4	0.9300
S1—O2	1.428 (6)	C5—C6	1.397 (11)
S1—N1	1.617 (6)	C5—H5	0.9300
S1—C8	1.792 (8)	C6—C7	1.457 (11)
O3—C7	1.215 (9)	C7—C8	1.540 (11)
N1—C1	1.408 (9)	C9—C10	1.442 (14)
N1—C9	1.486 (9)	C9—H9A	0.9700
C1—C6	1.388 (10)	C9—H9B	0.9700
C1—C2	1.418 (11)	C10—H10A	0.9600
C2—C3	1.380 (13)	C10—H10B	0.9600
C2—H2	0.9300	C10—H10C	0.9600
C3—C4	1.354 (14)		
			
O1—S1—O2	119.0 (4)	C1—C6—C5	119.6 (7)
O1—S1—N1	109.3 (4)	C1—C6—C7	123.8 (7)
O2—S1—N1	111.5 (3)	C5—C6—C7	116.6 (7)
O1—S1—C8	110.8 (4)	O3—C7—C6	123.7 (7)
O2—S1—C8	104.2 (4)	O3—C7—C8	119.1 (7)
N1—S1—C8	100.3 (3)	C6—C7—C8	117.3 (6)
C1—N1—C9	120.6 (6)	C7—C8—S1	108.0 (5)
C1—N1—S1	118.2 (5)	C7—C8—Br2	111.4 (5)
C9—N1—S1	121.1 (5)	S1—C8—Br2	110.3 (4)
C6—C1—N1	122.4 (7)	C7—C8—Br1	107.8 (5)
C6—C1—C2	118.7 (7)	S1—C8—Br1	109.4 (4)
N1—C1—C2	118.9 (7)	Br2—C8—Br1	109.9 (4)
C3—C2—C1	119.1 (8)	C10—C9—N1	114.5 (7)
C3—C2—H2	120.4	C10—C9—H9A	108.6
C1—C2—H2	120.4	N1—C9—H9A	108.6
C4—C3—C2	122.1 (8)	C10—C9—H9B	108.6
C4—C3—H3	119.0	N1—C9—H9B	108.6
C2—C3—H3	119.0	H9A—C9—H9B	107.6
C3—C4—C5	119.4 (8)	C9—C10—H10A	109.5
C3—C4—H4	120.3	C9—C10—H10B	109.5
C5—C4—H4	120.3	H10A—C10—H10B	109.5
C4—C5—C6	121.1 (8)	C9—C10—H10C	109.5
C4—C5—H5	119.5	H10A—C10—H10C	109.5
C6—C5—H5	119.5	H10B—C10—H10C	109.5
			
O1—S1—N1—C1	−167.9 (6)	C1—C6—C7—O3	−171.2 (8)
O2—S1—N1—C1	58.5 (7)	C5—C6—C7—O3	10.1 (12)
C8—S1—N1—C1	−51.4 (6)	C1—C6—C7—C8	8.3 (11)
O1—S1—N1—C9	16.6 (8)	C5—C6—C7—C8	−170.4 (7)
O2—S1—N1—C9	−117.0 (7)	O3—C7—C8—S1	139.1 (7)
C8—S1—N1—C9	133.1 (7)	C6—C7—C8—S1	−40.4 (8)
C9—N1—C1—C6	−160.8 (7)	O3—C7—C8—Br2	17.8 (10)
S1—N1—C1—C6	23.7 (10)	C6—C7—C8—Br2	−161.7 (6)
C9—N1—C1—C2	19.1 (11)	O3—C7—C8—Br1	−102.8 (8)
S1—N1—C1—C2	−156.4 (6)	C6—C7—C8—Br1	77.7 (7)
C6—C1—C2—C3	−0.3 (12)	O1—S1—C8—C7	173.0 (5)
N1—C1—C2—C3	179.8 (8)	O2—S1—C8—C7	−57.8 (6)
C1—C2—C3—C4	−1.5 (14)	N1—S1—C8—C7	57.7 (6)
C2—C3—C4—C5	1.0 (15)	O1—S1—C8—Br2	−65.0 (5)
C3—C4—C5—C6	1.4 (14)	O2—S1—C8—Br2	64.1 (4)
N1—C1—C6—C5	−177.6 (7)	N1—S1—C8—Br2	179.6 (4)
C2—C1—C6—C5	2.6 (11)	O1—S1—C8—Br1	55.9 (5)
N1—C1—C6—C7	3.7 (12)	O2—S1—C8—Br1	−174.9 (4)
C2—C1—C6—C7	−176.1 (8)	N1—S1—C8—Br1	−59.4 (4)
C4—C5—C6—C1	−3.2 (13)	C1—N1—C9—C10	65.8 (11)
C4—C5—C6—C7	175.6 (8)	S1—N1—C9—C10	−118.8 (8)

### Hydrogen-bond geometry (Å, °)

**Table:**

*D*—H···*A*	*D*—H	H···*A*	*D*···*A*	*D*—H···*A*
C2—H2···O1^i^	0.93	2.52	3.390 (10)	157
C9—H9A···O1	0.97	2.38	2.876 (11)	111

Symmetry codes: (i) *x*, −*y*+1/2, *z*+1/2.
